# Morphological and Molecular Evolution Are Not Linked in *Lamellodiscus* (Plathyhelminthes, Monogenea)

**DOI:** 10.1371/journal.pone.0026252

**Published:** 2011-10-12

**Authors:** Timothée Poisot, Olivier Verneau, Yves Desdevises

**Affiliations:** 1 UPMC Univ Paris 06, UMR 7232, Biologie Intégrative des Organismes Marins, Observatoire Océanologique, Banyuls-sur-Mer, France; 2 CNRS, UMR 7232, Biologie Intégrative des Organismes Marins, Observatoire Océanologique, Banyuls-sur-Mer, France; 3 UMR 5110 CNRS-UPVD, Centre de Formation et de Recherche sur les Environnements Méditerranéens, Université de Perpignan Via Domitia, Perpignan, France; Biodiversity Insitute of Ontario-University of Guelph, Canada

## Abstract

*Lamellodiscus* Johnston & Tiegs 1922 (Monogenea, Diplectanidae) is a genus of common parasites on the gills of sparid fishes. Here we show that this genus is probably undergoing a fast molecular diversification, as reflected by the important genetic variability observed within three molecular markers (partial nuclear 18S rDNA, Internal Transcribed Spacer 1, and mitonchondrial *Cytochrome Oxidase I*). Using an updated phylogeny of this genus, we show that molecular and morphological evolution are weakly correlated, and that most of the morphologically defined taxonomical units are not consistent with the molecular data. We suggest that *Lamellodiscus* morphology is probably constrained by strong environmental (host-induced) pressure, and discuss why this result can apply to other taxa. Genetic variability within nuclear 18S and mitochondrial *COI* genes are compared for several monogenean genera, as this measure may reflect the level of diversification within a genus. Overall our results suggest that cryptic speciation events may occur within *Lamellodiscus*, and discuss the links between morphological and molecular evolution.

## Introduction

Describing new species solely on the basis of their morphology is often not straightforward, and especially so for small-bodied organisms that display few morphological features on which to rely. A good illustration is highlighted in monogenean parasitic flatworms, where the main morphological structures used for species identification, namely the hard parts of the host attachment apparatus (haptor) and male copulatory organ, often require expert advice to discriminate closely related species, and although displaying phylogenetic conservatism in some genera [1], may display variations with environmental conditions [2]–[4] and host species [5], [6], eventually leading to speciation [7]. It is not clear whether morphological variation within a species should be linked to an ongoing speciation process, if it emerges as a combination of inter-individual variation and (potentially host-induced) polymorphism in the population, or involves any combination of these factors. In the specific case of monogeneans, the haptoral parts, because they are used by the parasite to attach to its host, are likely to be more strongly affected by phenotypic plasticity in generalist species (i.e. using several host species with varying gill characteristics [8]), even if this process appears to be limited [9]. It is now well described that species can engage in phenotype switching to cope with complex (in the case of parasites, multi-hosts) environments [10], which result in the coexistence of potentially different forms of the same species [11]. In this case, the existence of different morphotypes would not correspond to different species, rendering molecular evaluation of the taxonomic situation necessary. This problem is obviously more difficult to tackle when there are few characters on which identification can be conducted, and when these characters are directly under environmental control (as is the case for the hard haptoral parts of the monogeneans).

*Lamellodiscus* (Monogenea, Diplectanidae) are gill parasites of sparid fish throughout the world [12]. In the past ten years, over twelve new species have been described within this genus in Mediterranean and African fishes [13]–[19]. These species were described solely on the basis of very few morphological variations in comparison to previously known *Lamellodiscus* species. The morphological differences between recently described species and their already described counterparts are often tedious to observe in light microscopy, making them highly questionable. The difficulties in species assignment in *Lamellodiscus* were highlighted in previous molecular analyses, which showed that some species like *Lamellodiscus virgula* and *L. obeliae*, because of their high similarities in sequences coding for 18S and ITS1 (% differences are respectively 0 and 0.27), could be synonymous species [20]. A more striking example is *Furnestinia echeneis*, that belongs to the genus *Lamellodiscus* [21], despite its blaring morphological divergence from previously known *Lamellodiscus* species. Most notably, *F. echeneis* attachment apparatus only harbors one lamellodisc, instead of two for all other *Lamellodiscus* species. The above examples stress that morphology should not be viewed as a consistently reliable tool in systematic investigation, and recent studies showed how this conclusion applies for other monogeneans [22] and free-living animals [23].

Beyond the use of molecular data for species-assignment purposes, a recent study by Hansen and colleagues [24], looking for differences between the two monogeneans *Gyrodactylus thymalli* and *Gyrodactylus salaris*, revealed the existence of several lineages, unveiling a higher than expected diversity. Bakke and colleagues [25] reported a similar result, proposing that there could be as many as 20000 *Gyrodactylus* species, due to their fast ability to diverge both on molecular and morphological characters. Due to the fact that gyrodactylids pose severe economic problems in aquaculture, they have been more extensively studied than any other monogenean genera, which explain that few data are available for other monogenean genera.

In this study, we used three genetic markers, the 3′ extremity of the 18S rRNA gene, the Internal Transcribed Spacer 1, and approximately 300 base pairs within the first subunit of the mitochondrial Cytochrome C Oxidase I, *COI*, to estimate the level of divergence at the intra- and interspecific levels in *Lamellodiscus*. We focused on recently described species from the morphological group *ignoratus* [14] that are characterized by simple lateral dorsal bars in the haptor and an *en lyre* (made of two loosely bound hard parts resembling the shape of a lyre) male copulatory organ. Several features of *L. ignoratus* s.l. (*sensu lato*, i.e. the group comprised of *L. ignoratus* s.s., *L. falcus*, *L. neifari*, *L. confusus* and *L. diplodi*) make it a suitable group for such a study: its four taxa are discriminated by discrete morphological differences. Finally, these species occur on a limited number of sparid hosts: *Diplodus sargus*, *D. vulgaris*, *D. annularis*, *D. puntazzo*, *Lithognathus mormyrus* and *Salpa salpa*.

Our goals were **(i)** to assess the taxonomic status of these recently described *Lamellodiscus* species: *L. neifari, L. falcus, L. confusus, L. diplodi;* **(ii)** to check whether or not these species are closely related to *L. ignoratus* (*sensu stricto*, henceforth referred to as *L. ignoratus* s.s.), thus comparing the relative merits of morphological and molecular investigation of species status in this genus; and **(iii)** to evaluate the level of molecular diversity in *Lamellodiscus*, within and between species and discuss how it can assist in species assignment problems.

## Materials and Methods

### 1 Fish and parasite sampling

Fishes were sampled near Banyuls-sur-Mer (42°28′47N, 3°08′10E), by free diving. Two hosts species were collected, *Diplodus sargus* and *D. vulgaris*, as they are known to harbor several *Lamellodiscus* species belonging to the *L. ignoratus* s.l. subgroup [12]. Immediately after capture, fish were killed by a sharp blow on the top of the head, and dissected. Gills were removed, and examined at most 30 minutes after removal, under a light stereomicroscope (Olympus SZ61), to check for the presence of *Lamellodiscus*.

Parasites were isolated from the gills, and placed on a slide to be examined under light microscope (Olympus CX41, 400 times magnification). Species identification was carried out based on the shape of the opistohaptor and male copulatory organ [15]. Parasites were the preserved and stored individually in 96% ethanol before DNA extraction.

### 2 DNA extraction and amplification

DNAs were extracted from dried samples in a mixture of 70 µl of Chelex (100 mg/ml) and 15 µl of Proteinase K (10 mg/ml) at 55 °C for one hour. Reactions were then stopped at 100 °C for 15 minutes and kept at 4 °C until used.

Three markers were used in our analysis: the 3′ terminal fragment of the 18S rDNA (18S), the Internal Transcribed Spacer 1 (ITS1) and partial mitochondrial gene Cytochrome Oxidase I (*COI*). Until now, only the 18S had been used for phylogenetic analysis in *Lamellodiscus* [20], [26], [21].

The 18S-ITS1 fragment was amplified in one round with primers L7 (forward, 5′-TGATTTGTCTGGTTTATTCCGAT-3′) and IR8 (reverse, 5′-GCTAGCTGCGTTCTTCATCGA-3′) as designed by Verneau and colleagues [27] and Šimková and colleagues [28] while *COI* was amplified with primers LCO1P (forward, 5′-TTTTTTGGGCATCCTGAGGTTTAT-3′) and HCox1P2 (reverse, 5′-TAAAGAAAGAACATAATGAAAATG-3′), after Littlewood and colleagues [29]. PCR were performed using the following cycles: 6 minutes at 95°C, then 35 cycles as follows: 1 minute at 95°C, 1 minute at 48°C, and 2 minutes at 72°C. A final elongation was conducted for 10 minutes at 72°C. PCR fragments were run in 1% agarose gels and purified using Nucleospin Extract II Gel extraction kit (Macherey-Nagel). They were sent to Macrogen Inc. (Korea) for sequencing. Sequences for this study were deposited in GenBank with numbers EU259028 to EU259032 and JF427625 to JF427661.

### 3 Distance computation and phylogenetic analysis

Due to the difficulty to align ITS1 even within a single genus, the following analyses were done on *COI* and *18S* only. GenBank [30] was first queried to retrieve 18S and *COI* sequences from monogeneans (species for which at least 3 sequences were available were included). ClustalW2 [31] was used to align all sequences for each marker with default settings, using the alignment of *Lamellodiscus* species as a reference (for both *18S* and *COI*). The ambiguously aligned parts were removed using Gblocks [32], [33], which retained 473 unambiguous positions out of 537 in the original 18S sequences. Uncorrected pairwise distances (excluding indels) were computed using the *dist.dna* function of the *APE* package [34] for R 2.9.0 [35]. Numbers of sequences by genus and species are listed in Tables 1 and 2.

**Table 1:** Mean pairwise distances (m.p.d.) for the 245 bp long fragment of the *COI* gene in several monogenean taxa.

Clade	Order	Rank	Sample size	m.p.d.
*Euryhaliotrema grandis*	M	isolate grap	5	
*E. grandis*	M	isolate gram	8	
*E. grandis*	M	isolate grah	4	0.0032
*E. grandis*	M	isolate gral	9	0.0067
*Haliotrema aurigae*	M	species	16	0.0070
*Gyrodactylus lavareti*	M	species	4	0.0081
*G. arcuatus*	M	species	7	0.0085
*Wetapolystoma almae*	P	species	3	0.0122
*G. salaris*	M	species	16	0.0153
*Lamellodiscus furcosus*	M	species	3	0.0327
*E. grandis*	M	species	27	0.0510
*G. lucii*	M	species	9	0.0514
*Protopolystoma xenopodi*	P	species	5	0.0688
*L. neifari*	M	unclear	2	0.0696
*Protopolystoma simplicis*	M	species	3	0.0860
*Protopolystoma* spp.	P	genus	10	0.1312
*Gyrodactylus* spp.	M	genus	38	0.1596
Polystomatidae	P	family	28	0.1766
*Lamellodiscus* spp.	M	genus	11	0.1988

Legend for column Order: P is for Polyopisthocotylea, M for Monopisthocotylea. m.p.d: mean of pairwise distances. Species for which at least 3 sequences were available were included in the analysis.

**Table 2:** Mean pairwise distances for the 641 bp long fragment of the 18S ribosomal DNA in several monogenean taxa.

Clade	Order	Rank	Sample size	m.p.d
*Dactylogyrus crucifer*	M	species	3	
*D. vistulae*	M	species	3	
*Pseudodactylogyrus* spp.	M	genus	17	0.0017
*Lamellodiscus neifari*	M	unclear	4	0.0027
*Dactylogyrus* spp.	M	genus	61	0.0238
*L. ignoratus* s.l.	M	LITU	17	0.0246
*Polystomoides* spp.	P	genus	8	0.0251
*Lamellodiscus* spp.	M	genus	46	0.0476
*Gyrodactylus salaris*	M	species	156	0.0514
*G. thymalli*	M	species	31	0.0832
*Gyrodactylus* spp.	M	genus	341	0.1175
Polystomatidae	P	family	86	0.3371

**Figure 1:**
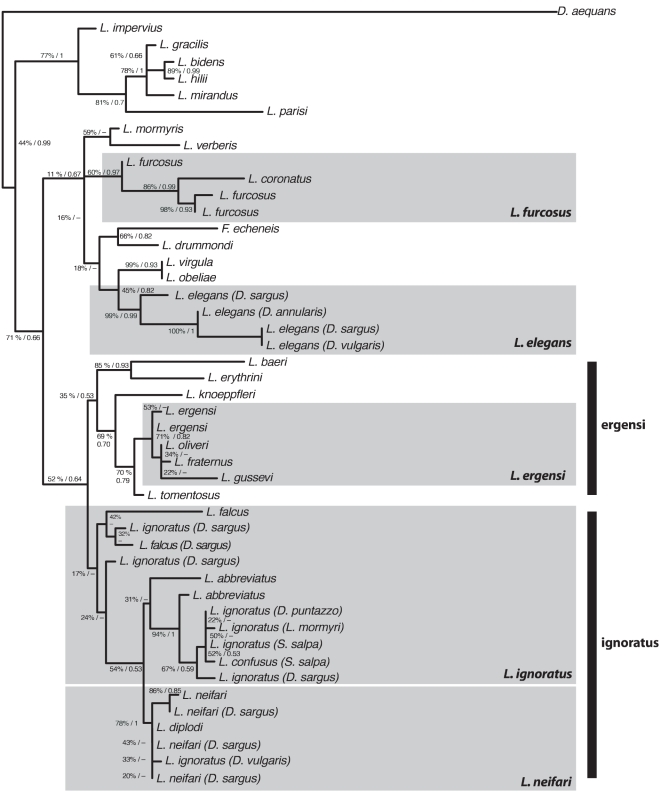
Phylogeny of several *Lamellodiscus* species obtained by maximum likelihood and Bayesian inference. As topologies obtained with both reconstruction methods gave congruent topologies and similar branch lengths, the most resolved tree, obtained by maximum likelihood, was retained and is presented here. Bootstrap values (1000 replicates) and posterior probabilities (>0.5. Dashes correspond to values <0.5) are indicated at each node. The clusters of individuals for which the alignment of ITS1 was possible are outlined in grey boxes. Thick black lines indicate ergensi and ignoratus groups.

See Table 1 for legend. The group formed by *L. ignoratus* s.l. (Figure 1) was given the status of litu *sensu* Pleijel and Rouse [62].

Due to the difficulty of obtaining enough sequences of *COI* in *Lamellodiscus*, the phylogenetic reconstruction was computed on the 18S fragment only. Evolutionary models were tested using ModelTest [36] and selected with regard to their AIC score, using PAUP* 4.0b10 [37]. Trees were inferred using two probabilistic approaches: maximum likelihood with a non-parametric bootstrap validation using PhyML [38] (using a GTR model with 49% of invariant sites and a Gamma shape parameter of 0.46), and Bayesian inference (using MrBayes 3.1.2 [39], [40], using 2 runs of 4 chains during 2.10^6^ generations, a burnin value of 25% of the saved trees, sampled every 100 generations; convergence was assessed using Tracer v. 1.5 [41], the average standard deviation of split frequencies was checked to be less than 0.01, and the potential scale reduction factors at the end of the runs were less than 1.01 for both the model parameters and the bipartitions in the consensus trees). The trees were rooted using *Diplectanum aequans* [42]. Each time the phylogenetic pattern obtained casted doubt upon the taxonomic status of a group of individuals, ITS1 sequences were manually aligned to help in determining whether they belong to the same species, as it has been previously suggested that ITS1 could be aligned within but not between *Lamellodiscus* species [20], [8]. However, given that this criterion deserves a more formal investigation, ITS1 is used along with other criteria such as genetic distance and phylogenetic pattern to assess species status.

## Results

### 1 Phylogeny of *Lamellodiscus*

Our updated *Lamellodiscus* phylogeny (Figure 1–the ML version is presented, as both reconstruction methods gave congruent topologies), using the 3′ end of 18S ribosomal DNA carries new information regarding the previous phylogeny obtained by Desdevises and colleagues [42], using the same portion of the 18S ribosomal DNA (but based on fewer species and using only one individual per species). The *L. ignoratus* s.l. group is not supported, with a bootstrap value of 17% for its most basal node (PP<0.5). Within this group, individuals from several putative species (both previously known and recently described from morphology) cluster together. Moreover, individuals from the same species are not clustered in this tree (for example, the sequence obtained for *L. coronatus* clusters in between the sequences obtained for *L. ignoratus*), which can be due to the fact that this part of the tree is overall poorly supported. However, so as to gain further insight on the species status of such groups, we checked that the ITS1 sequences could be aligned. The clusters of individuals for which this alignment was possible are outlined in grey boxes in Fig. 1, suggesting that these groups might have taxonomical relevance, but were incorrectly attributed to the various species of the *ignoratus* group. *COI* was not used in phylogenetic analyses due to the difficulties of getting a large number of sequences, as no standard amplification protocol for this marker exists.

### 2 Intraspecific and interspecific pairwise distances

From the partial 18S, the mean of uncorrected pairwise distances between all sequences available for *Lamellodiscus* is 5.7%. The distance between *L. elegans* (AF294956) and *L. parisi* (AY038198), the most divergent sequences, is 9.2%. For the least divergent sequences, *L. fraternus* (AY038191) and *L. ergensi* (AY038190), the distance is 0.6%. Within *L. ignoratus* s.l. individuals (n = 17), we were able to align all ITS1 sequences in two groups (containing 11 and 6 individuals), suggesting that all specimens within these groups belong to the same species (named *L. ignoratus* and *L. neifari* on Fig. 1), thus highlighting incongruences between morphological and molecular identifications. The mean distance for 18S of *L. ignoratus* s.l. considered as a single taxonomic entity is 2.46%.

Pairwise distances for the markers *COI* and 18S are listed in Tables 1 and 2, respectively. Variability within the genus *Lamellodiscus* is the highest observed in our sample for the *COI* gene. Concerning the 18S, the genus *Lamellodiscus* is more variable than any other monogenean genera, except *Gyrodactylus*, as indicated by the higher genetic distances. The amount of variability correlates with the taxonomic level for *COI* (i.e. isolates are less variable than species, and species less than genera), but not for *18S*. It should be noted that this result is likely to be influenced by the fact that sampling effort was stronger on some monogenean genera, and that should be kept in mind when interpreting these observations.

## Discussion

### 1 Cryptic speciation in *Lamellodiscus*?

Because of their strong potential for diversification, monogeneans are a promising model to study biodiversity issues [43]. This assumption is supported by the estimation of 25000 monogenean species by Rohde [44]. Note that Bakke and colleagues [25] estimated 20000 species in the genus *Gyrodactylus* only, making it one of the most speciose animal genera known. *Gyrodactylus* is one of the most studied monogenean genera, because of the impact of some species in aquaculture [45], [46], but data for other monogeneans are lacking. Here we suggest that *Lamellodiscus*, compared to other monogeneans, is characterized by a high molecular diversity at both intraspecific and intrageneric level. Among the two main morphologically defined groups, *ergensi* was poorly supported by the molecular phylogeny, while *ignoratus* forms an unsupported cluster of individuals. In addition, the situation within each putative group is complex: phylogenetic support is weak within each group, where distinct morphs are found, among which some are close to each other from molecular data (grey boxes in Fig. 1). Within the *ergensi* group, the small sample size precludes any meaningful observation. Within the *ignoratus* group, while supported nodes exist, they are not compatible with groups that could be delineated using morphological characters. For example, individuals from *L. ignoratus* s.s. are interspersed in the *L. ignoratus* s.l. group, and one of the *L. abbreviatus* individuals is outside a strongly supported clade containing the other *L. abbreviatus* isolates. Based on partial 18S rRNA gene sequences, several species appear to be either not monophyletic (e.g. *L. ignoratus, L. ergensi*) or invalid (such as *L. coronatus* which clusters within *L. furcosus* individuals), which suggests that *Lamellodiscus* could be either more diversified than expected, or that there is a gap between the morphological characterization of species and their evolutionary relatedness (these two propositions not being mutually exclusive). This claim is supported by the magnitude of pairwise distances observed, particularly at the lower taxonomic levels, between *Lamellodiscus* individuals.

Three main factors can be invoked to explain the putative high ability for diversification in monogeneans, reflected in the high molecular and morphological diversity observed in *Lamellodiscus*. First, habitat heterogeneity is likely to be greater for small-bodied organisms (such as *Lamellodiscus*), thus favoring their diversification [47]–[49]. Poulin [50] observed this pattern in monogenean ectoparasites : there are far more small-bodied fish monogeneans than large-bodied ones. Second, monogeneans have a direct life cycle. Life-cycle complexity has been suggested to affect speciation rates in parasites [43]. Within Platyhelminthes, which share a common origin of the parasitic lifestyle, the Monogenea is the only group in which an adaptive evolutionary radiation has occurred [51]. Because of their small body size and direct life cycle, monogeneans have an important potential for diversification [28]. Finally, the genus *Lamellodiscus* appears to be composed of more species with a wide host range than any other monogenean genera, which has been suggested to add some molecular variability [8] and potential for speciation [6], [52]–[54].

Relying on morphology alone led previous researchers to consider as belonging to the same species some morphs that were found in different clades in our molecular phylogeny. This is emphasized by the situation of *L. ignoratus* s.l., where none of the described species receives support based on the molecular data. This situation could be interpreted in two ways. First, most species in the *ignoratus* group might be paraphyletic. By paraphyletic, we mean that within a cluster of related individuals belonging to the same species, one or several individuals from another species branch out. The existence of paraphyletic species has already been observed both for free-living [55] and symbiotic [56]–[58] taxa. In our sample, some pairwise genetic distances between individuals from different species (e.g. 0.6% between *L. fraternus* and *L. ergensi* based on partial 18S rDNA) were found to be lower than some intra-specific distances. This situation strongly suggests that two or more species are not a monophylum [59]. Second, *Lamellodiscus* may contain more species that our current estimation. Several studies aimed to characterize new species in this genus in the last few years [13], [15], [17], based on very small morphological variations. However, these recently described species are not easily differentiated from others (neither from morphological nor molecular analyses), and according to the molecular evidence presented here, it might be more conservative to consider that they are *species inquirenda* (i.e. species of doubtful identity).

The fact that the recently described species are not necessarily valid must not lead us to lump all *L. ignoratus* s.l. individuals into a single species. Indeed, pairwise distances within *L. ignoratus* s.l. are higher than for any other species pairs (Table 1), and comparable to those observed in other genera (the partial 18S rDNA diversity of *L. ignoratus* s.l. is comparable to what was observed between *Dactylogyrus* spp. and between *Polystomoides* spp., see Table 2), and while they do not correspond to what could be delineated based on morphological character, there are some supported clades in the *ignoratus* group. This result suggests that several species could exist in the *L. ignoratus* s.l. group, but due to a high diversity and putative cryptic speciation, an intensive sampling is still needed to gather enough data to detect them.

The precise knowledge of which taxa are species is crucial, because species, contrary to higher level taxa, are not only an outcome of evolution, but are also directly involved in evolutionary processes [60], [61]. We face the same problem in *Lamellodiscus*: our current view of the evolution of this genus [42] was inferred according to what we thought to be species; if what we called species was rather an assemblage of dissociated taxonomical units, some of the mechanisms thought to act in *Lamellodiscus* evolution (such as radiation by host switch followed by speciation) need to be re-evaluated in the light of revised species delineation and an updated phylogeny. Before assessing intraspecific and intrageneric genetic diversity, it is important to be sure which taxa are given the species status. In such a situation, it could be useful to use the Least-inclusive taxonomic unit (LITU) concept [62], that is considering several individuals as forming a clade, without making further assumptions about the taxonomic position of this clade. Our results suggest that the *ignoratus* group is highly diversified, and is likely to be formed by several OTUs; we suggest to give this group the status of LITU, and to wait for further investigation to determine its exact taxonomic status. According to these results, our view of the taxonomy and, consequently, of the evolution of *Lamellodiscus* needs to be reassessed.

### 2 Phylogeny and morphology seem to be unlinked in *Lamellodiscus*

The molecular variability (as approximated by the pairwise distance at several taxonomical depths) in *Lamellodiscus* was compared to what was found in other monogenean genera. For *COI*, *Lamellodiscus* shows the most important interspecific distances; for 18S, *Lamellodiscus* is nearly twice as variable as *Dactylogyrus*, but less than half as variable as *Gyrodactylus*, thought to be the most variable monogenean genus [63]. Despite this important molecular variability, however, there are very little clearly distinguishable morphologies within the genus *Lamellodiscus*. Amine and Euzet [14] defined two morphological groups in this genus, named ignoratus (formed by *L. ignoratus* s.l., *L. fraternus*, *L. knoeppfleri* and *L. erythrini*) and ergensi (formed by *L. ergensi*, *L. sanfilippoi*, *L. kechemirae* and *L. baeri*). Our results (Figure 1) are congruent with this classification, with two notable exceptions: *L. knoeppfleri*, *L. fraternus* and *L. erythrini* were found to belong to the ergensi group. According to our phylogeny, the ignoratus group is only formed of the taxa belonging to *L. ignoratus* s.l. Within each group, however, there is no link between morphological features and phylogenetic position, mostly because none of the individuals of a single putative species cluster together. A similar situation was reported by Hay and colleagues [64]. They observed that the tuatara, *Sphenodon punctatus*, while being a living fossil (its morphology is strikingly similar to the fossil specimens), and having a slow metabolism, a long generation time, and a slow rate of reproduction, is the species having the highest rate of molecular evolution observed amongst vertebrates. Hence, a high molecular divergence is not necessarily linked to important morphological changes, and the assumption that rates of molecular and morphological evolution are inherently correlated [65] is likely to be untrue in certain genera, which could be the case in *Lamellodiscus*.

Given that we were able to align the ITS1 of several individuals, we are able to make suggestions as to the species status of some morphotypes. We found molecular evidences that, despite some morphological divergences on the shape of the hard parts and copulatory organs, *L. furcosus* and *L. coronatus* form a single species (that we call *L. furcosus*). The ITS1 of *L. ergensi*, *L. oliveri*, *L. fraternus* and *L. gussevi* can be aligned, suggesting that all of these morphotypes should be considered as a single species, that we call *L. ergensi*. The individuals belonging to *L. neifari* and *L. diplodi*, as well as some *L. ignoratus* individuals, display enough ITS1 similarity to allow their sequences to be aligned. As for other species, we suggest that these species are invalid, and that only *L. neifari* should be retained. The situation is similar for *L. falcus*, *L. ignoratus*, *L. abbreviatus* and *L. confusus*. We suggest that these morphotypes belong to the *L. ignoratus* species, and that the others are invalid. More data (e.g. other genes) are needed to confirm this pattern, as shown by the lack of support from 18S sequences in this part of the tree. However, we did not rule out the possibility that diversification is acting within these species, which is likely given the important genetic divergence observed within *Lamellodiscus*. Owing to the relatively low support of some nodes in the phylogeny (Fig. 1), we suggest that *Lamellodiscus* may be highly diverse, and our understanding of their taxonomical status will benefit from an increased genetic sampling.

The apparent discrepancies between morphological features and molecular phylogeny could be explained by the strong selective pressures parasites have to cope with. Morphology in *Lamellodiscus* (and in most monogeneans) is mainly studied by looking at the sclerotized parts of the haptor and the male copulatory organ, which is a putative factor in the initiation of intra-host speciation events amongst monogeneans [66], [28]. In *Lamellodiscus*, as in most (if not all) Monogenan genera, no information is available about the degree of morphological dissimilarity that must be achieved between two shapes of male copulatory organs to trigger a reproductive isolation event leading to speciation. However, all species in the ignoratus and ergensi groups do harbor an *en lyre* male copulatory organ, with some variation between species [14]; this supports the hypothesis that these groups share a direct common ancestor. The monophyly of both ignoratus and ergensi groups is strongly supported by morphological characters, such as the split of lateral dorsal bars, and this result is supported by our phylogeny (modulo the position of *L. knoeppfleri*, *L. fraternus* and *L. erythrini*, which may be cases of convergent evolution, or merely reflect the difficulty to determine what constitutes a “character” [67]). It seems that, while the strong (e.g. number of lamellodiscs, number of pieces in the lateral bars) differences between organ shapes are linked to the taxonomic position of species, the small differences (e.g. shape or width of some parts of dorsal and ventral hooks) are not indicative of a speciation process [68]. However, molecular data suggest that some features (non-split bars in some species within the ergensi group) may display an evolutionary convergence, under environmental (that is, host-induced) pressure.

### 3 Genetic diversity in *Lamellodiscus* and other monogeneans

During this study, we assessed mean uncorrected genetic pairwise distances based on two molecular markers (the 5′ end of the 18S gene, and about 300 base pairs within *COI*) on several monogenean genera. For the *COI* gene (Table 1), it seems that the mean pairwise distance is an appropriate reflection of the taxonomic position: intra-specific uncorrected pairwise genetic distances range from 0% (in *Euryhaliotrematoides grandis* individuals from the same isolate) to 8% (between individuals of *Protopolystoma simplicis*); intra-generic distances range from 13% (for *Protopolystoma* spp.) to 19% (for *Lamellodiscus*). The notable exception to this pattern is the Polystomatidae family, with an intra-family distance of 17%. However, the latter result may be due to the fact that few sequences are available to cover a whole family [69], thus potentially decreasing the mean distance, and emphasizing the need to gather more genetic data at broad taxonomical scales. Another explanation is that larger bodied monogeneans might be less speciose than smaller bodied organisms. Another explanation is that chelonian polystomes arose very early, in the Lower Triassic, namely 200 Million years ago [70]. This may explain large divergences observed between species of different genera. For the *18S* gene (Table 1), however, the pattern of correspondence between taxonomic position and mean pairwise distance is lost. While some species display very few variations (the sequences we retrieved for *Dactylogyrus crucifer* and *D. vistulae* showed no differences), others (such as *G. salaris* and *G. thymalli* thought to be a single species [71]) harbor a level of intra-specific divergence comparable or superior to the one found in the *Lamellodiscus* and *Gyrodactylus* genera. Altogether, these results indicate that analyses of pairwise genetic distances to assess taxonomic status, although used in diverse biological systems [72]–[74], should be conducted cautiously as not all markers display the same behavior of congruence between genetic distance and taxonomical rank.

It seems that the evolutionary rate of some markers, such as 18S rDNA, is lineage specific (e.g. *Gyrodactylus* seems to evolve faster than *Lamellodiscus*, itself evolving faster than *Dactylogyrus*), whereas in other markers, such as *COI*, mean distance correlates with taxonomic position. These results can be due to different evolutionary rate in these markers (*COI* is mitochondrial and coding, 18S is nuclear and structural), but may also be linked to sampling effort: where some genera have undergone an important sampling effort (e.g. *Gyrodactylus*), few molecular data are yet available for others or, when they are, they often come from a single study, often limited to a single geographic area despite the broad geographical distribution of *Lamellodiscus* [75]. The question of whether the current amount of available data allows us to capture the majority of the genetic diversity in monogeneans remains pending. Moreover, it is likely that the lifestyle of the various taxa will matter in determining the genetic diversity; for example, how viviparous and egg laying monogeneans differ in this extent is yet to be investigated.

### 4 Concluding remarks

This study suggests that the degree of variability displayed by the different markers used here is impacted by the taxonomic position of the group investigated. Here, this variability is linked to the taxonomic position for *COI*, but not for 18S. In comparison to the important genetic variability displayed by *Lamellodiscus*, there is a relative morphological conservatism, suggesting the action of environmental (host-induced) selection pressures on the shape of several haptoral parts.

## References

[1] Vignon M, Pariselle A, Vanhove MPM (2011). Modularity in attachment organs of African *Cichlidogyrus* (Platyhelminthes, Monogenea, Ancyrocephalidae ) reflects phylogeny rather than host specificity or geographic distribution.. Biological Journal of the Linnean Society.

[2] Ergens R, Gelnar M (1985). Experimental verification of the effect of temperature on the size of hard parts of opisthaptor of *Gyrodactylus katharineri* Malmberg, 1964 (Monogenea).. Folia Parasitologica.

[3] Mo TA (1991). Variations of opisthaptoral hard parts of *Gyrodactylus salaris* Malmberg, 1957 (Monogenea: Gyrodactylidae) on parr of Atlantic Salmon *Salmo salar* L. in laboratory experiments.. Systematic Parasitology.

[4] Dávidová M, Jarkovský J, Matejusová I, Gelnar M (2005). Seasonal occurrence and metrical variability of *Gyrodactylus rhodei* Zitnan 1964 (Monogenea, Gyrodactylidae).. Parasitology research.

[5] Mo TA (1993). Seasonal variations of the opisthaptoral hard parts of *Gyrodactylus derjavini* Mikailov, 1975 (Monogenea: Gyrodactylidae) on brown trout *Salmo trutta* L. parr and Atlantic salmon *S. salar* L. parr in the River Sandvikselva, Norway.. Systematic Parasitology.

[6] Poisot T, Desdevises Y (2010). Putative speciation events in *Lamellodiscus* (Monogenea: Diplectanidae) assessed by a morphometric approach.. Biological Journal of the Linnean Society.

[7] Bueno-Silva M, Boeger WA, Pie MR (2011). Choice matters: incipient speciation in *Gyrodactylus corydori* (Monogenoidea: Gyrodactylidae).. International Journal for Parasitology.

[8] Kaci-Chaouch T, Verneau O, Desdevises Y (2008). Host specificity is linked to intraspecific variability in the genus *Lamellodiscus* (Monogenea).. Parasitology.

[9] Matejusová I, Koubková B, Gelnar M, Cunningham CO (2002). *Paradiplozoon homoion* Bychowsky & Nagibina, 1959 versus *P. gracile* Reichenbach-Klinke, 1961 (Monogenea): two species or phenotypic plasticity?. Systematic Parasitology.

[10] Poisot T, Bever JD, Nemri A, Thrall PH, Hochberg ME (2011). A conceptual framework for the evolution of ecological specialization.. Ecology Letters.

[11] Libby E, Rainey PB (2011). Exclusion rules, bottlenecks and the evolution of stochastic phenotype switching.. Proceedings of the Royal Society B: Biological Sciences: rspb..

[12] Euzet L, Combes C, Caro A (1993). A checklist of Monogenea of Mediterranean fish.. Second International Symposium on Monogenea.

[13] Neifar L, Euzet L, Oliver G (2004). *Lamellodiscus* (Plathelminthes, Monogenea, Diplectanidae) nouveaux parasites branchiaux des poissons marins du genre *Pagrus* (Teleostei, Sparidae).. Zoosystema.

[14] Amine F, Euzet L (2005). Deux espèces nouvelles du genre *Lamellodiscus* Johnston & Tiegs, 1922 (Monogenea: Diplectanidae) parasites de Sparidae (Teleostei) des côtes de l′Algérie.. Systematic Parasitology.

[15] Amine F, Euzet L, Kechemir-Issad N (2007). *Lamellodiscus theroni* sp. nov. (Monogenea, Diplectanidae), a gill parasite from *Diplodus puntazzo* (Teleostei, Sparidae) from the Mediterranean Sea.. Acta Parasitologica.

[16] Amine F, Euzet L, Kechemir-Issad N (2006). Description de deux nouvelles espèces de *Lamellodiscus* Johnston & Tiegs, 1922 (Monogenea: Diplectanidae) du groupe morphologique “ignoratus”, parasites de *Diplodus sargus* et *D. vulgaris* (Teleostei: Sparidae).. Systematic Parasitology.

[17] Amine F, Euzet L, Kechemir-Issad N (2007). Description de *Lamellodiscus confusus* n. sp. (Monogenea: Diplectanidae), parasite de Sarpa salpa (Teleostei: Sparidae).. Parasite.

[18] Amine F, Neifar L, Euzet L (2006). *Lamellodiscus sanfilippoi* n. sp. (Monogenea, Diplectanidae) parasite from the gills of Diplodus sargus (Teleostei, Sparidae) in Mediterranean Sea.. Parasite.

[19] Diamanka A, Neifar L, Pariselle A, Euzet L (2011). *Lamellodiscus* (Monogenea: Diplectanidae) parasites of *Dentex macrophthalmus* (Teleostei: Sparidae) from the North Atlantic coast of Africa, with a redescription of *L. dentexi* Aljoshkina, 1984, and description of three new species.. Folia Parasitologica.

[20] Desdevises Y, Jovelin R, Jousson O, Morand S (2000). Comparison of ribosomal DNA sequences of *Lamellodiscus* spp. (Monogenea, Diplectanidae) parasitising *Pagellus* (Sparidae, Teleostei) in the North Mediterranean Sea: species divergence and coevolutionary interactions.. International Journal for Parasitology.

[21] Desdevises Y (2001). The phylogenetic position of *Furnestinia echeneis* (Monogenea, Diplectanidae) based on molecular data: a case of morphological adaptation?. International Journal for Parasitology.

[22] Perkins EM, Donnellan SC, Bertozzi T, Chisholm LA, Whittington ID (2009). Looks can deceive: molecular phylogeny of a family of flatworm ectoparasites (Monogenea: Capsalidae) does not reflect current morphological classification.. Molecular Phylogenetics and Evolution.

[23] Havermans C, Nagy ZT, Sonet G, Broyer C De, Martin P (2010). Incongruence between molecular phylogeny and morphological classification in amphipod crustaceans: a case study of Antarctic lysianassoids.. Molecular Phylogenetics and Evolution.

[24] Hansen H, Bakke TA, Bachmann L (2007). Mitochondrial haplotype diversity of *Gyrodactylus thymalli* (Platyhelminthes; Monogenea): extended geographic sampling in United Kingdom, Poland, and Norway reveals further lineages.. Parasitology research.

[25] Bakke T, Harris PD, Cable J (2002). Host specificity dynamics: observations on gyrodactylid monogeneans.. International Journal for Parasitology.

[26] Desdevises Y, Morand S, Legendre P (2002). Evolution and determinants of host specificity in the genus *Lamellodiscus* (Monogenea).. Biological Journal of the Linnean Society.

[27] Verneau O, Renaud F, Catzeflis F (1997). Evolutionary relationships of sibling tapeworm species (Cestoda) parasitizing teleost fishes.. Molecular Biology and Evolution.

[28] Simková A, Morand S, Jobet E, Gelnar M, Verneau O (2004). Molecular phylogeny of congeneric monogenean parasites (*Dactylogyrus*): a case of intrahost speciation.. Evolution.

[29] Littlewood DT, Rohde K, Clough KA (1997). Parasite speciation within or between host species? Phylogenetic evidence from site-specific polystome monogeneans.. International Journal for Parasitology.

[30] Benson DA, Karsch-Mizrachi I, Lipman DJ, Ostell J, Sayers EW (2010). GenBank.. Nucleic acids research.

[31] Thompson JD, Higgins DG, Gibson TJ (1994). CLUSTAL W: improving the sensitivity of progressive multiple sequence alignment through sequence weighting, position-specific gap penalties and weight matrix choice.. Nucleic Acids Research.

[32] Castresana J (2000). Selection of conserved blocks from multiple alignments for their use in phylogenetic analysis.. Molecular Biology and Evolution.

[33] Talavera G, Castresana J (2007). Improvement of phylogenies after removing divergent and ambiguously aligned blocks from protein sequence alignments.. Systematic Biology.

[34] Paradis E, Claude J, Strimmer K (2004). APE: Analyses of Phylogenetics and Evolution in R language.. Bioinformatics.

[35] R Development Core Team (2008). R: A Language and Environment for Statistical Computing.

[36] Posada D, Crandall KA (1998). MODELTEST: testing the model of DNA substitution.. Bioinformatics.

[37] Swofford DL (2002). PAUP*: phylogenetic analysis using parsimony (* and other methods).

[38] Guindon S, Gascuel O (2003). A simple, fast, and accurate algorithm to estimate large phylogenies by maximum likelihood.. Systematic Biology.

[39] Huelsenbeck JP, Ronquist F (2001). MRBAYES: Bayesian inference of phylogenetic trees.. Bioinformatics.

[40] Ronquist F, Huelsenbeck JP (2003). MrBayes 3: Bayesian phylogenetic inference under mixed models.. Bioinformatics.

[41] Rambault A, Drummond AJ (2007). http://beast.bio.ed.ac.uk/Tracer.

[42] Desdevises Y, Morand S, Jousson O, Legendre P (2002). Coevolution between *Lamellodiscus* (Monogenea: Diplectanidae) and Sparidae (Teleostei): the study of a complex host-parasite system.. Evolution.

[43] Poulin R, Morand S (2000). The diversity of parasites.. Quarterly Review of Biology.

[44] Rohde K (2005). Marine Parasitology..

[45] Shinn AP, Gibson DI, Sommerville C (2001). Morphometric discrimination of *Gyrodactylus salaris* Malmberg (Monogenea) from species of *Gyrodactylus* parasitising British salmonids using novel parameters.. Journal of Fish Diseases.

[46] Olstad K, Cable J, Robersten G, Bakke TA (2006). Unpredicted transmission strategy of *Gyrodactylus salaris* (Monogenea: Gyrodactylidae): survival and infectivity of parasites on dead hosts.. Parasitology.

[47] Marzluff JM, Dial KP (1991). Life history correlates of taxonomic diversity.. Ecology.

[48] Maurer BA, Brown JH, Rusler RD (1992). The Micro and Macro in Body Size Evolution.. Evolution.

[49] Fenchel T (1993). There Are More Small Than Large Species?. Oikos.

[50] Poulin R (1996). The evolution of body size in the Monogenea: the role of host size and latitude.. Canadian Journal of Zoology.

[51] Park J-K, Kim K-H, Kang S, Kim W, Eom KS (2007). A common origin of complex life cycles in parasitic flatworms: evidence from the complete mitochondrial genome of *Microcotyle sebastis* (Monogenea: Platyhelminthes).. BMC Evolutionary Biology.

[52] Vrba ES (1987). Ecology in relation to speciation rates: some case histories of Miocene-Recent mammal clades.. Evolutionary Ecology.

[53] García-Robledo C, Horvitz CC (2011). Experimental demography and the vital rates of generalist and specialist insect herbivores on native and novel host plants.. Journal of Animal Ecology.

[54] Matsubayashi KW, Kahono S, Katakura H (2011). Divergent host plant specialization as the critical driving force in speciation between populations of a phytophagous ladybird beetle.. Journal of Evolutionary Biology.

[55] Talbot SL, Shields GF (1996). Phylogeography of brown bears (*Ursus arctos*) of Alaska and paraphyly within the Ursidae.. Molecular Phylogenetics and Evolution.

[56] Brown JM, Pellmyr O, Thompson JN, Harrison RG (1994). Phylogeny of Greya (Lepidoptera: Prodoxidae ), Based on Nucleotide Sequence Variation in Mitochondrial Cytochrome Oxidase I and II: Congruence with Morphological Data interactions.. Molecular Biology and Evolution.

[57] Lymbery AJ, Thompson RC (1996). Species of *Echinococcus*: pattern and process.. Parasitology Today.

[58] Ellis JT, Morrison DA, Liddell S, Jenkins MC, Mohammed OB (1999). The genus *Hammondia* is paraphyletic.. Parasitology.

[59] Harrison G, Howard DJ, Berlocher SH (1992). Linking Evolutionary Pattern and Process.. Endless forms: Species & speciation.

[60] de Queiroz K, Donoghue MJ (1988). Phylogenetic systematics and the species problem.. Cladistics.

[61] Luckow M (1995). Species concepts: assumptions, methods, and applications.. Systematic Botany.

[62] Pleijel F, Rouse GW (2000). Least-inclusive taxonomic unit: a new taxonomic concept for biology.. Proceedings of the Royal Society B: Biological Sciences.

[63] Hansen H, Bakke TA, Bachmann L (2007). DNA taxonomy and barcoding of monogenean parasites: lessons from *Gyrodactylus*.. Trends in parasitology.

[64] Hay J, Subramanian S, Millar C (2008). Rapid molecular evolution in a living fossil.. Trends in Genetics.

[65] Omland KE (1997). Correlated Rates of Molecular and Morphological Evolution.. Evolution.

[66] Morand S, Simkova A, Matejusova I, Plaisance L, Verneau O (2002). Investigating patterns may reveal processes: evolutionary ecology of ectoparasitic monogeneans.. International Journal for Parasitology.

[67] Donoghue MJ, Sanderson MJ, Hall BK (1994). Complexity and homology in plants.. Homology: the hierarchical basis of comparative biology.

[68] Pouyaud L, Desmarais E, Deveney M, Pariselle A (2006). Phylogenetic relationships among monogenean gill parasites (Dactylogyridea, Ancyrocephalidae) infesting tilapiine hosts (Cichlidae): systematic and evolutionary implications.. Molecular Phylogenetics and Evolution.

[69] Verneau O, Palacios C, Platt T, Alday M, Billard E (2011). Invasive species threat: parasite phylogenetics reveals patterns and processes of host-switching between non-native and native captive freshwater turtles.. http://dx.doi.org/10.1017/S0031182011000333.

[70] Verneau O, Bentz S, Sinnappah ND, Preez L du, Whittington I (2002). A view of early vertebrate evolution inferred from the phylogeny of polystome parasites (Monogenea: Polystomatidae).. Proceedings of the Royal Society B: Biological Sciences.

[71] Huyse T, Buchmann K, Littlewood DTJ (2008). The mitochondrial genome of *Gyrodactylus derjavinoides* (Platyhelminthes: Monogenea) – a mitogenomic approach for *Gyrodactylus* species and strain identification.. Gene.

[72] Bradley RD, Baker RJ (2001). A test of the genetic species concept: cytochrome-b sequences and mammals.. Journal of Mammalogy.

[73] Söller R, Warnke K, Saint-Paul U, Blohm D (2000). Sequence divergence of mitochondrial DNA indicates cryptic biodiversity in *Octopus vulgaris* and supports the taxonomic distinctiveness of *Octopus mimus* (Cephalopoda: Octopodidae).. Marine Biology.

[74] Beaumont M, Barratt EM, Gottelli D, Kitchener AC, Daniels MJ (2001). Genetic diversity and introgression in the Scottish wildcat.. Molecular Ecology.

[75] Justine JL, Briand MJ (2010). Three new species, *Lamellodiscus tubulicornis* n. sp., *L. magnicornis* n. sp. and *L. parvicornis* n. sp. (Monogenea: Diplectanidae) from *Gymnocranius* spp. (Lethrinidae: Monotaxinae) off New Caledonia, with the proposal of the new morphological group ‘tubulicornis’ within *Lamellodiscus* Johnston & Tiegs, 1922.. Systematic Parasitology.

